# Unveiling the “Glycolysis-Lactate-Lactylation” Axis: A Novel Target in Ischemic Cerebrovascular Disease

**DOI:** 10.1007/s12264-026-01622-5

**Published:** 2026-04-06

**Authors:** Yi Xie, Mengmeng Dai, Rui Liu, Ying Li, Hao Yan, Jiacheng Li, Xuantong Liu, Zhiyuan Yu, Shabei Xu, Wei Wang, Xiang Luo

**Affiliations:** 1 Department of Neurology, Tongji Hospital, Tongji Medical College, Huazhong University of Science and Technology, Wuhan, 430030 China; 2 Hubei Key Laboratory of Neural Injury and Functional Reconstruction, Huazhong University of Science and Technology, Wuhan, 430030 China; 3 Hubei Stroke Clinical Research Center, Wuhan, 430030 China; 4 Key Laboratory of Neurological Diseases of the Chinese Ministry of Education, School of Basic Medicine, Tongji Medical College, Huazhong University of Science and Technology, Wuhan, 430030 China

**Keywords:** Ischemic cerebrovascular disease, Glycolysis, Lactate, Lactylation, Metabolic reprogramming, Neuroinflammation

## Abstract

Ischemic cerebrovascular disease involves complex interactions between metabolic reprogramming and epigenetic regulation. Recent studies indicate that enhanced glycolysis and lactate accumulation under hypoxic conditions not only maintain cell viability by supplying energy but also participate in disease regulation through lactylation. Lactylation regulates gene expression, immune polarization, metabolic enzyme activity, and angiogenesis through epigenetic remodeling. In ischemic cerebrovascular disease, lactylation exerts dual roles: on one hand, it can induce neuronal death, exacerbate neuroinflammation, and form a vicious metabolic cycle; on the other hand, it can influence immune cell function and gene expression, thereby exerting neuroprotective effects. Accordingly, targeting lactate metabolism or lactylation-modifying enzymes holds considerable therapeutic potential. Nevertheless, the spatiotemporal regulation, synergistic effects, and broader physiopathological implications of lactylation warrant further in-depth investigation. This review systematically summarizes the role of the “glycolysis-lactate-lactylation” axis in the pathogenesis of ischemic cerebrovascular disease, and discusses its potential as a therapeutic target.

## Glycolysis and Lactate Production

**Fig. 1:**
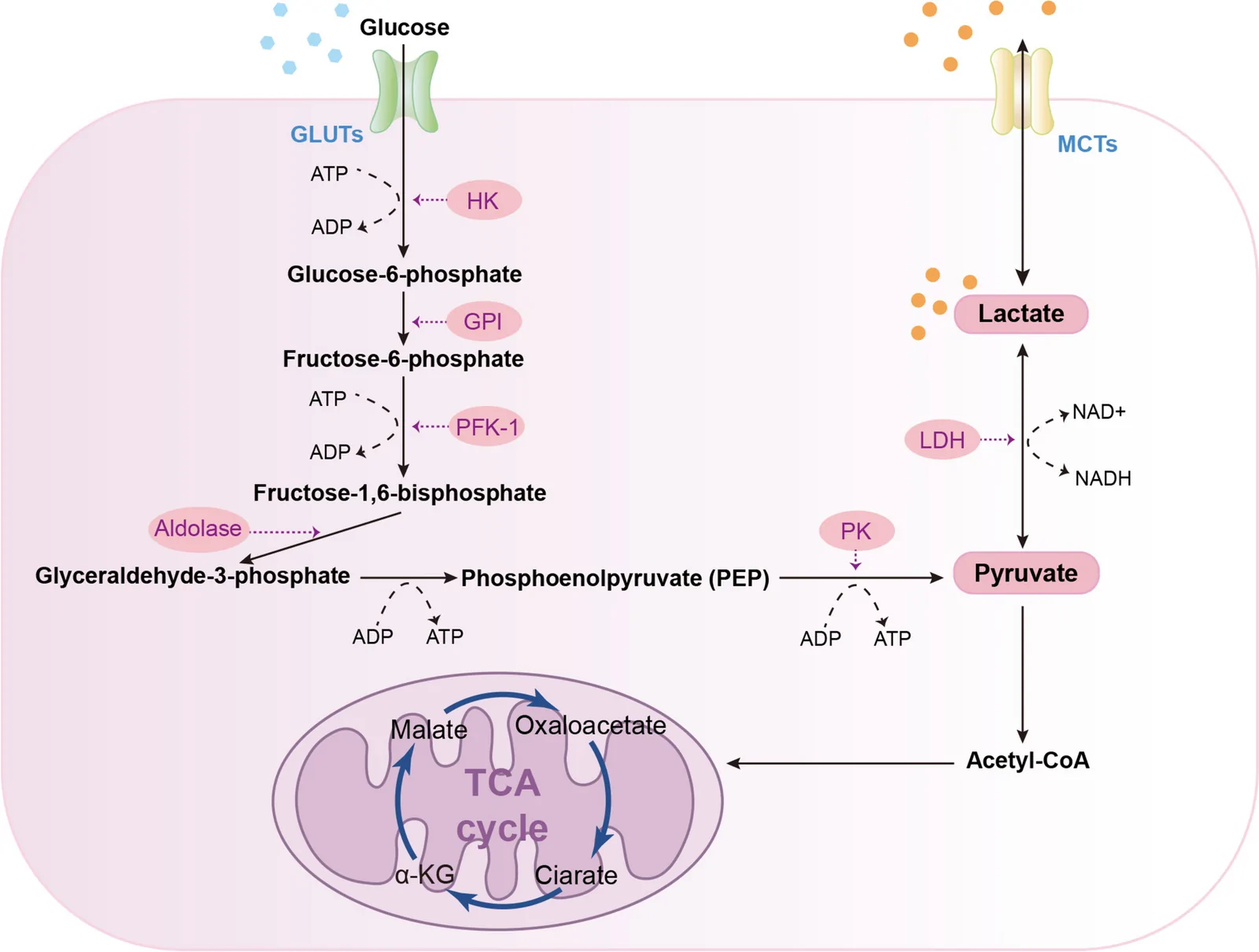
The process of glycolysis and lactate production. After entering the cell *via* GLUTs, glucose is phosphorylated by HK to form glucose-6-phosphate. Subsequently, GPI isomerizes glucose-6-phosphate to fructose-6-phosphate, which is further metabolized to pyruvate through a series of enzymatic reactions. Under normal conditions, pyruvate is further converted to acetyl-CoA by pyruvate dehydrogenase, entering the TCA cycle to produce large amounts of ATP. However, under hypoxic conditions, pyruvate is reduced to lactate by LDH. In addition, lactate can be transported across the cell membrane by a variety of MCTs. Abbreviations: HK, Hexokinase; GPI, Glucose-6-phosphate isomerase; PFK-1, Phosphofructokinase-1; PK, Pyruvate kinase.

Glucose metabolism is a complex process primarily involving glycolysis, further metabolism of pyruvate, and the tricarboxylic acid (TCA) cycle (Fig. 1). Under aerobic conditions, glucose enters cells *via* glucose transporters (GLUTs), is phosphorylated by hexokinase to glucose-6-phosphate, and is metabolized through glycolysis to yield pyruvate [1, 2]. Subsequently, pyruvate is transported into the mitochondria, where it is converted into acetyl-CoA by pyruvate dehydrogenase (PDH). Acetyl-CoA then enters the TCA cycle, producing reducing equivalents in the form of nicotinamide adenine dinucleotide (NADH) and flavin adenine dinucleotide (FADH₂). These molecules fuel oxidative phosphorylation in the inner mitochondrial membrane, where electrons are transferred to oxygen, generating water and large amounts of adenosine triphosphate (ATP) [3]. However, under hypoxic conditions, pyruvate remains in the cytoplasm and is reduced to lactate by lactate dehydrogenase (LDH), a process termed anaerobic glycolysis. In this reaction, LDH oxidizes NADH to NAD⁺, and the regeneration of NAD⁺ sustains glycolysis, enabling each glucose molecule to yield a net gain of two ATP molecules [3]. Through these metabolic pathways, glucose provides a sustained energy source for cells, ensuring the physiological functions of organisms under varying oxygen conditions.

Although lactate production can rapidly provide energy, its ATP output is significantly lower than that of aerobic respiration. Additionally, the accumulation of lactate lowers intracellular pH, impairing the function of enzymes and proteins [4]. In severe cases, it can even lead to systemic acidosis, such as during profound shock or global hypoperfusion [5, 6]. Therefore, lactate must be rapidly cleared from tissues and circulation through metabolism. Nevertheless, anaerobic glycolysis retains essential physiological functions. First, under hypoxic conditions, it can rapidly supply energy [7, 8]. Second, for certain specialized cells lacking mitochondria or with high proliferative activity, glycolysis serves as the primary energy source. Furthermore, lactate can enhance the activation of acetyl-CoA carboxylase (ACC), thereby promoting fatty acid synthesis [9]. Additionally, glycolytic intermediates serve as precursors for the synthesis of lipids and amino acids.

## Lactylation

Although lactate has long been regarded as a metabolic byproduct of glycolysis under hypoxia, emerging evidence indicate that its intracellular accumulation not only supports energy compensation but also directly influences gene expression and cellular function through a novel epigenetic mechanism—lactylation. This discovery elevates lactate from “terminal metabolite” to “signaling regulator”, offering a fresh perspective on its multifaceted roles in hypoxic conditions.

### Definition of Lactylation

Lactylation, a newly identified post-translational modification (PTM) involving the covalent binding of lactate to lysine residues (Kla), has attracted considerable attention since its discovery in 2019. Specifically, it was found that lactylation of histone 3 lysine 18 (termed H3K18la) in pro-inflammatory M1 macrophages is associated with the upregulation of M2-like genes, promoting a phenotypic shift toward the anti-inflammatory M2 macrophage state [10]. Recent studies increasingly underscore the broader biological significance of lactylation, extending the role of lactate beyond metabolism to the regulation of gene expression, epigenetics, immune responses, and metabolic control [11]. Accumulating evidence further links lactylation to the pathogenesis of cancer, neurological and cardiovascular diseases, and inflammation [12, 13].

### Mechanisms of Lactylation

**Fig. 2:**
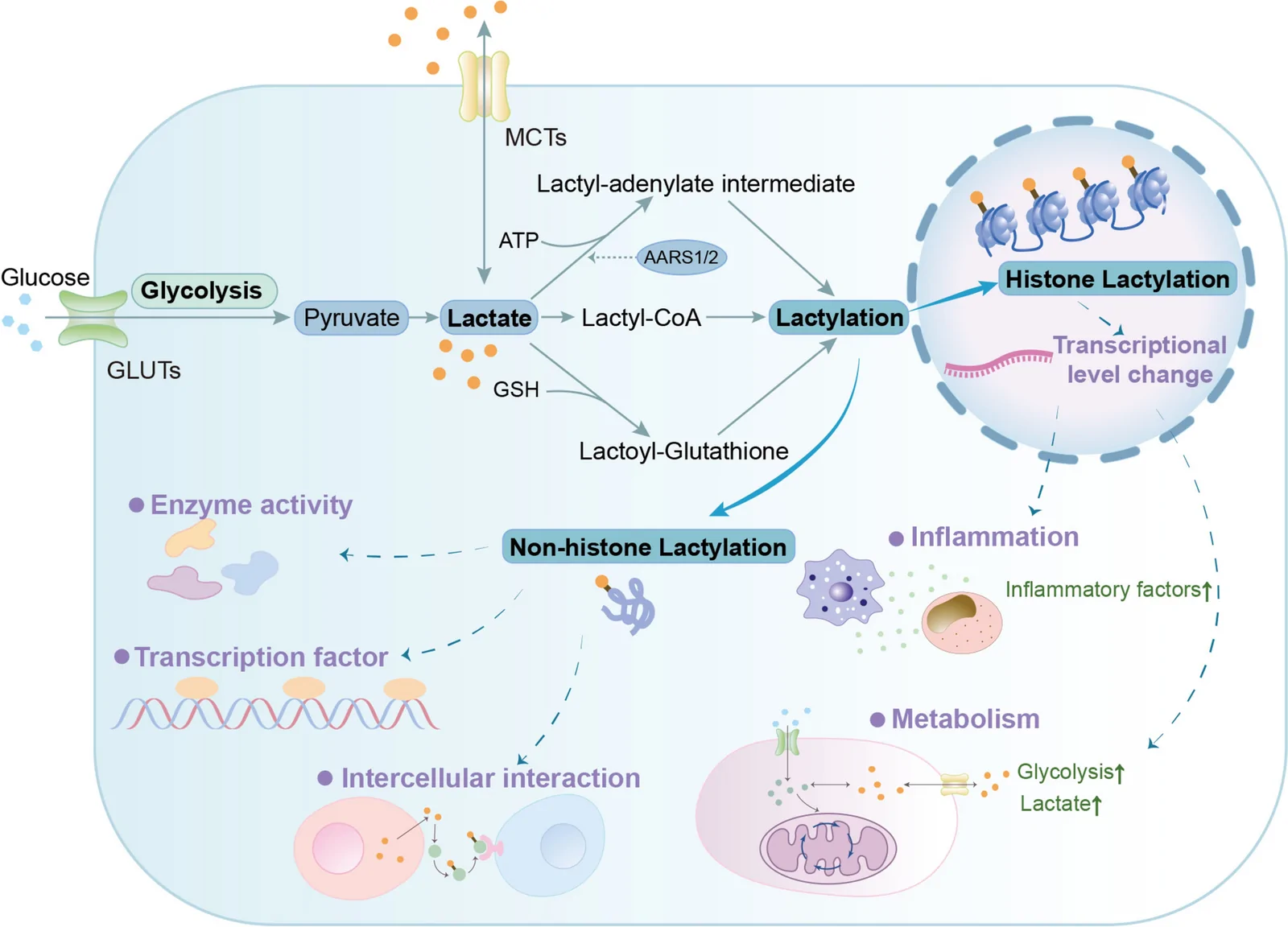
The mechanisms, classifications, and functions of lactylation. Glucose enters the cell *via* GLUTs and undergoes glycolysis to produce pyruvate, which is then converted to lactate. Next, lactate can either be processed through the lactyl-CoA enzymatic pathway or directly utilized by AARS1/2 to complete lactylation. Additionally, lactate can generate lactyl-glutathione, which non-enzymatically reacts with lysine to form lactylation. Lactylations include histone lactylation and non-histone lactylation. Histone lactylation modulates gene transcription, inflammation, and metabolism, while non-histone protein lactylation also has important effects on regulating enzyme activity, transcription factor function, and signal transduction. Abbreviations: GSH, glutathione.

The fundamental principle of lactylation involves the covalent attachment of lactate molecules to lysine residues on proteins (Fig. 2). Lactylation primarily occurs through enzyme-catalyzed processes. Upon intracellular accumulation, lactate can be converted into lactyl-CoA, which donates the lactyl group to lysine residues in reactions catalyzed by the “writer” enzymes such as p300/CREB-binding protein (CBP) and histone acetyltransferase HBO1 [14–16]. Additionally, aminoacyl-tRNA synthetase 1/2 (AARS1/2) can directly utilize lactate and ATP to attach lactate to lysine residues independently of lactyl-CoA [17]. Lactylation may also arise through non-enzymatic mechanisms, primarily *via* the methylglyoxal metabolic pathway, which generates lactylglutathione [18, 19]. The metabolite then directly reacts with lysine residues on proteins to achieve this modification [19, 20]. Together, these findings suggest that lactylation can be mediated by diverse enzymatic and non-enzymatic pathways, reflecting its broad regulatory potential.

### Lactylations of Different Proteins

Lactylations have been identified in both histone and non-histone proteins, influencing gene expression, cellular metabolism, signal transduction, and immune responses, thereby contributing to numerous physiological and pathological conditions (Fig. 2).

#### Histone Lactylations

Histone lactylation mainly occurs on lysine residues, altering chromatin structure and gene transcription [15]. During the late phase of M1 macrophage polarization, lactylation of histones promotes the expression of wound healing-related genes, such as arginase 1 (Arg1), thereby driving the transition of cells toward a more M2-like homeostatic phenotype [10]. In tumor cells, HBO1-mediated H3K9la modification promotes the activation of key signaling pathways and the initiation and progression of tumorigenesis [21]. Meanwhile, histone lactylation plays a significant role in metabolic regulation. In pancreatic ductal adenocarcinoma (PDAC), enhanced glycolysis elevates lactate production, which in turn promotes H3K18la modification and activates the transcription of mitotic checkpoint regulators TTK protein kinase and BUB1 mitotic checkpoint serine/threonine kinase B. This establishes a glycolysis-H3K18la-TTK/BUB1B feedback loop that accelerates PDAC progression [22]. Furthermore, histone lactylation is closely related to immune regulation and inflammatory responses. Notably, it can be enriched in CD8^+^ T cells, marking effector function-related genes such as Gzmb and Ifngr1, thereby promoting inflammatory responses [23]. In glioblastoma (GBM), monocyte-derived macrophages (MDMs) possess high glycolytic activity, and their metabolic product, lactate, promotes the expression of the anti-inflammatory cytokine IL-10 through histone lactylation, thereby inhibiting T cell activity and modulating immune responses [24].

#### Non-histone Lactylations

In recent years, non-histone protein lactylation has also garnered considerable attention. Unlike histone lactylation, which primarily affects chromatin structure and gene expression, non-histone lactylation regulates a broader range of cellular functions, including enzyme activity, signaling pathways, cytoskeletal stability, and intercellular interactions.

These modifications occur in proteins distributed across the nucleus, cytoplasm, mitochondria, ribosomes, and plasma membrane. For instance, lactylation of adenylate kinase 2 (AK2) inhibits its activity and promotes the proliferation and metastasis of hepatocellular carcinoma cells [25]. Similarly, lactylation can regulate the activity of glycolytic enzymes, affecting cellular energy metabolism [26]. Furthermore, non-histone lactylation is able to modulate protein-protein interactions and subcellular localization by altering protein conformation and charge, thereby influencing intercellular interactions. In sepsis, lactylation of high-mobility group box 1 (HMGB1) facilitates its translocation from the nucleus to the cytoplasm and subsequent secretion *via* exosomes. The released HMGB1 increases endothelial permeability and exacerbates inflammation, underscoring its critical role in sepsis pathogenesis [16]. Moreover, non-histone lactylation can impact the function of transcription factors and cofactors, thereby regulating gene expression. In the tumor microenvironment, lactate enters cells *via* monocarboxylate transporter 1 (MCT1) and promotes lactylation of hypoxia-inducible factor-1α (HIF-1α). Lactylated HIF-1α acts as a transcriptional enhancer, binding to target gene promoters to drive transcription, and thereby promoting tumor angiogenesis and metabolic reprogramming [27]. Similarly, Chai et al. discovered that transcription factor Yin Yang-1 (YY1) can undergo lactylation catalyzed by p300 in retinal microglia. Excessive lactylation of YY1 at the K183 site significantly increases the transcription of fibroblast growth factor 2 (FGF2), driving oxygen-induced retinal neovascularization [28].

## Glycolysis, Lactate Production, and Lactylation in Diseases

Lactate in the human body originates from multiple sources, with glycolysis being the primary pathway. In addition, glutamine catabolism contributes to lactate production by supplying carbon backbones [9], while fatty acid β-oxidation generates acetic acid, which can be further metabolized into lactate under hypoxic conditions or during metabolic reprogramming [29]. However, an isotopic labeling study has demonstrated that the contributions of glutamine and acetate are relatively minor, with lactate predominantly derived from glucose metabolism [29]. That is to say, although lactate originates from diverse sources, anaerobic glycolysis remains the predominant pathway. Elevated lactate production drives lactylation, establishing the “glycolysis-lactate-lactylation” axis that underpins diverse pathophysiological processes of diseases.

### Glycolysis, Lactate Production, and Lactylation in Cancer

In cancer, dysregulation of the “glycolysis-lactate-lactylation” axis forms a complex metabolic network. As early as 1926, Otto Warburg described the “Warburg effect”, whereby cancer cells preferentially convert glucose into lactate *via* glycolysis, even under aerobic conditions, instead of relying on mitochondrial oxidative phosphorylation [30]. This metabolic reprogramming leads to significant accumulation of lactate in cancer cells, subsequently triggering enhanced lactylation. This epigenetic regulation contributes to tumor progression and offers potential therapeutic targets [31].

**Fig. 3:**
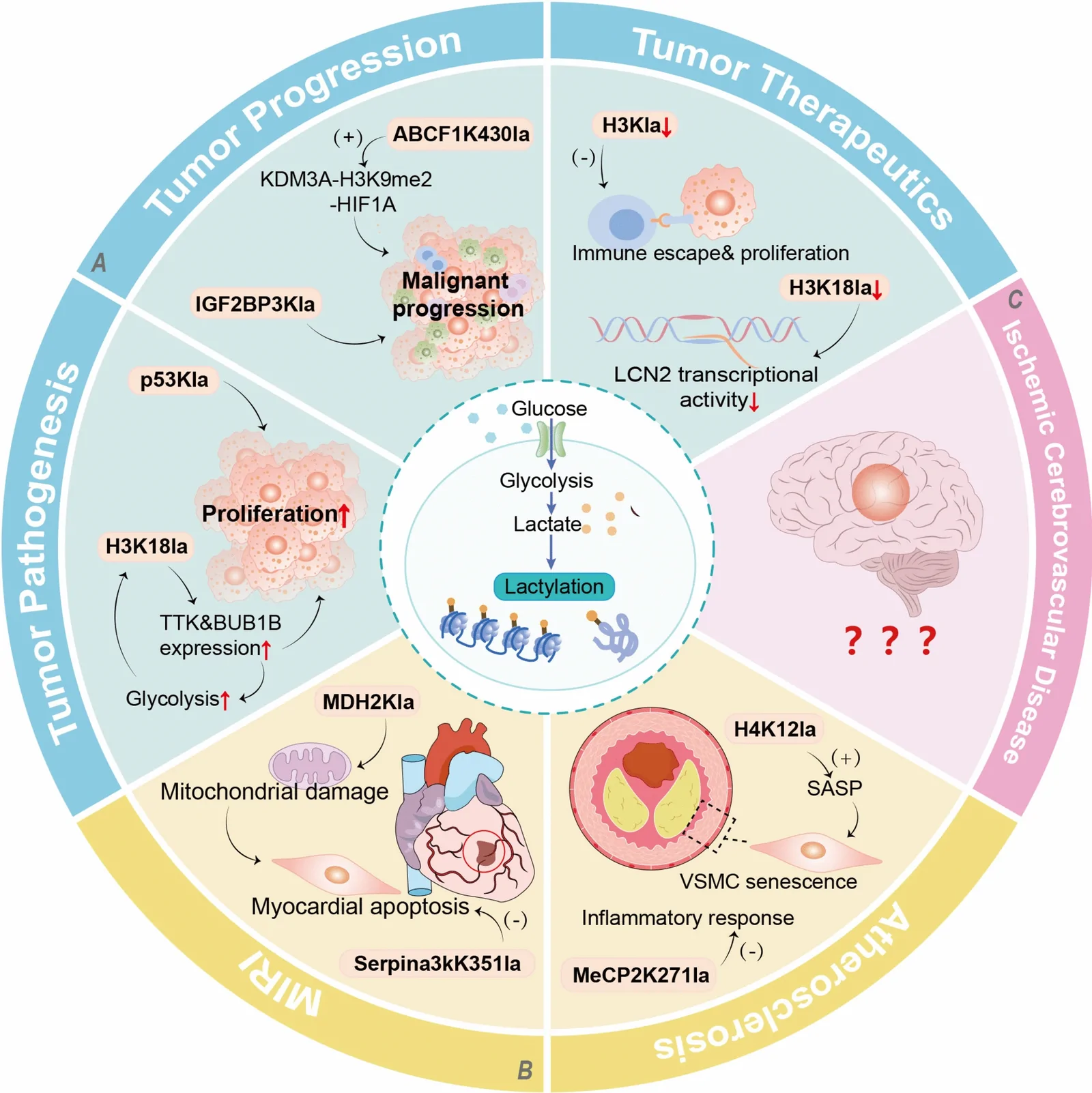
The roles of lactylation in cancer and cardiovascular diseases. Lactylation is implicated in the pathogenesis and progression of tumors and has an important impact on tumor therapeutics. Beyond oncology, lactylation also plays a significant role in cardiovascular diseases, including myocardial ischemia-reperfusion injury and atherosclerosis, by modulating key pathological processes. Moreover, emerging evidence highlights its important regulatory functions in ischemic cerebrovascular diseases. Symbols: (+) = Activation, (-) = Inhibition.

Lactylation is involved in tumorigenesis by inhibiting tumor suppressors and reprogramming metabolism. For example, tumor-derived lactate suppresses p53 function through AARS1-mediated lactylation, leading to uncontrolled proliferation [17]. In pancreatic ductal adenocarcinoma (PDAC), elevated histone lactylation, particularly H3K18la, is able to activate transcription of TTK and BUB1B to promote the occurrence of PDAC. These factors also upregulate p300, further enhancing glycolysis and forming a positive feedback loop that exacerbates the malignant progression of PDAC [22] (Fig. 3A).

Lactylation also drives tumor progression by regulating signaling pathways, altering metabolic activity, and remodeling the tumor microenvironment. In hepatocellular carcinoma (HCC), non-histone lactylation of ABCF1 at lysine 430 (ABCF1-K430la) facilitates its nuclear translocation and binding to the KDM3A promoter, activating the KDM3A-H3K9me2-HIF-1α signaling axis and thereby enhancing tumor growth [32]. Moreover, lactylation regulates the metabolism of cells and creates a favorable microenvironment for tumor progression. In another HCC study, the lactylation of insulin-like growth factor 2 mRNA binding protein 3 (IGF2BP3) enhances its binding to phosphoenolpyruvate carboxykinase 2 (PCK2) and nuclear factor erythroid 2-related factor 2 (NRF2) mRNA, promoting serine metabolic reprogramming and glycolysis. This forms a positive feedback loop *via* s-adenosylmethionine (SAM)-mediated m6A modification, reshaping the metabolic and epigenetic state of the tumor microenvironment, ultimately leading to enhanced antioxidant defense and levofloxacin resistance [33] (Fig. 3A).

The in-depth study of lactylation not only clarifies the molecular basis of tumor development but also uncovers promising avenues for treatment. Recent work showed that aminoacyl-tRNA synthetase 1/2 (ACSS1/2) interacts with lysine acetyltransferase 2A (KAT2A), which uses lactyl-CoA to modify histone H3. This process activates Wnt/β-catenin, NF-κB, and PD-L1 pathways, promoting brain tumor growth and immune evasion [34]. Targeting the “ACSS2–KAT2A” axis thus provides a dual-intervention strategy with greater efficacy than single-target therapies. In bladder cancer, circXRN2, a type of circular RNA, was found to suppress H3K18 lactylation by regulating the Hippo pathway, thereby downregulating the oncogene lipocalin 2 (LCN2), and inhibiting tumor progression [35] (Fig. 3A).

### Glycolysis, Lactate Production, and Lactylation in Cardiovascular Diseases

#### Myocardial Ischemia-Reperfusion Injury (MIRI)

During MIRI, impaired mitochondrial oxidative phosphorylation under hypoxia forces cardiomyocytes to rely on glycolysis, leading to increased lactate production. While this shift transiently sustains energy supply, prolonged lactate accumulation induces acidosis and calcium dysregulation, ultimately impairing cardiomyocyte function [20, 36]. In MIRI, Han She *et al.* found that increased lactylation of malate dehydrogenase 2 (MDH2) inhibits its activity, disrupts mitochondrial metabolism, and promotes cardiomyocyte apoptosis [37]. Dexmedetomidine has been shown to downregulate MDH2 lactylation, thereby blocking ferroptosis and mitochondrial damage, and reducing cardiomyocyte death [37]. Moreover, lactylation of serpina3k at lysine 351, secreted by cardiac fibroblasts, enhances protein stability and enables paracrine protection of cardiomyocytes during ischemia-reperfusion injury. By inhibiting the wingless/int (WNT) signaling pathway, and activating reperfusion injury salvage kinase (RISK) and survival activated factor enhancement (SAFE) pathways, serpina3k lactylation reduces reperfusion-induced apoptosis and alleviates myocardial injury [38]. Overall, these observations establish lactylation as a key regulator in MIRI pathogenesis and highlight its potential as a therapeutic target (Fig. 3B).

#### Atherosclerosis

The “glycolysis-lactate-lactylation” axis also plays a critical role in atherosclerosis. Endothelial cells within atherosclerotic plaques exhibit enhanced glycolysis, which promotes neovascularization and increases plaque instability [39–41]. Remarkably, lactate accumulation not only drives inflammation but also augments lactylation, thereby influencing disease progression. In aortic tissues of patients with atherosclerosis, tumor necrosis factor receptor-associated protein 1 (TRAP1) is upregulated and induces histone H4K12 lactylation (H4K12la), which accelerates the senescence of vascular smooth muscle cells (VSMC) and plaque formation [42]. VSMC-specific knockout of TRAP1 evidently alleviates atherosclerosis in mice [42]. Additionally, in 2024, Liangqi Chen *et al*. revealed for the first time that exercise improves atherosclerosis through the methyl-CpG-binding protein 2 (MeCP2) K271 lactylation-H3K36me3/runt-related transcription factor 1(RUNX1) axis [43]. Specifically, exercise-induced lactate promotes MeCP2 lactylation, suppresses mitogen-activated protein kinase (MAPK) pathway, thereby reducing inflammatory factors and adhesion molecules, and attenuating plaque inflammation [43] (Fig. 3B).

### Glycolysis, Lactate Production, and Lactylation in Ischemic Cerebrovascular Disease

Lactylation has broad implications in cancer and cardiovascular diseases, where its therapeutic potential is increasingly recognized. Emerging evidence suggests that the “glycolysis-lactate-lactylation” axis also contributes to ischemic cerebrovascular disease, though research in this field remains limited. Given the shared hypoxia-driven pathophysiology with cardiovascular disorders, this axis may play a pivotal role in disease development. The following sections will comprehensively discuss the roles of the “glycolysis-lactate-lactylation” axis in ischemic cerebrovascular disease (Fig. 3C).

## Glycolysis in Ischemic Cerebrovascular Disease

Ischemic cerebrovascular disease is a group of conditions caused by the blockage of cerebral blood vessels, leading to ischemia and hypoxia in brain tissue. Its pathogenesis is complex and involves metabolic alterations in various cell types [44]. In ischemic cerebrovascular disease, glycolysis is a critical metabolic pathway activated under hypoxic conditions to provide energy. Although less efficient than aerobic metabolism, glycolysis can rapidly supply the necessary energy to maintain basic cellular functions in the short term. However, the accumulation of lactate, a byproduct of glycolysis, can lead to local tissue acidosis and impair normal cellular function [45]. In ischemic cerebrovascular disease, metabolic shifts primarily occur in neurons, astrocytes, microglia, macrophages, and endothelial cells.

Brain tissue exhibits an exceptionally high demand for oxygen and glucose, particularly in neurons. Due to their substantial energy requirements and dense mitochondrial content, neurons predominantly depend on aerobic metabolism. Moreover, the expression of key glycolytic enzymes is significantly lower in neurons compared to other cells with high glycolytic activity, such as tumor cells and glial cells [46]. Thus, neurons have a poor glycolytic capacity and often need other cells to transport lactate to them to maintain energy metabolism [47]. Therefore, when cerebral ischemia occurs, neuronal energy metabolism rapidly shifts from aerobic metabolism to glycolysis to sustain basic cellular functions. The ATP depletion leads to the failure of ion pumps, such as Na⁺/K⁺-ATPase, thereby disrupting transmembrane ionic gradients and inducing neuronal excitotoxicity [48]. Additionally, ischemia triggers massive glutamate release, which overactivates N-methyl-D-aspartate receptors (NMDARs) and α-amino-3-hydroxy-5-methyl-4-isoxazolepropionic acid receptors (AMPARs), leading to Ca^2^⁺ influx. Calcium overload in neurons activates proteases, phospholipases, and other enzymes, damaging cellular structures and inducing apoptosis [49]. Ischemia also triggers pathological cascades, including the generation of reactive oxygen species (ROS) [50] and activation of inflammatory responses [51], which further exacerbate neuronal injury. Consequently, neurons are among the earliest cell types to be affected by ischemia and hypoxia.

Hypoxia also profoundly impacts astrocyte metabolism. Under hypoxic conditions, the oxidative capacity of astrocytes decreases, leading to more pyruvate being converted into lactate, significantly increasing the lactate-to-pyruvate ratio [52]. Additionally, studies have shown that hypoxia induces the overexpression of pyruvate dehydrogenase kinase 4 (PDK4) in astrocytes, causing the phosphorylation and inactivation of pyruvate dehydrogenase (PDH), further inhibiting pyruvate entry into the TCA cycle [53, 54]. Consequently, mitochondrial oxidative phosphorylation is limited, and glycolytic activity is markedly enhanced in astrocytes. Moreover, hypoxia also accelerates glycogenolysis in astrocytes. Specifically, it activates astrocytic glycogen phosphorylase, which catalyzes the breakdown of glycogen into glucose-1-phosphate, subsequently entering glycolysis to produce lactate [54, 55]. Since glycolysis is oxygen-independent and glycogen is primarily stored in astrocytes [56], astrocytic glycolysis theoretically becomes the main energy-producing pathway during cerebral ischemia, potentially supporting astrocyte activation post-ischemia [57]. Furthermore, lactate generated through astrocytic glycolysis can be shuttled to neurons *via* MCTs. Once transported, lactate is converted to pyruvate by lactate dehydrogenase 1 (LDH1) and subsequently enters the TCA cycle, thereby serving as a preferred energy substrate for neurons under hypoxic conditions [58, 59]. Therefore, in ischemic cerebrovascular disease, astrocytes adapt to hypoxia by upregulating glycolysis and supplying lactate to neurons [52], thereby playing a crucial role in maintaining brain homeostasis and neuroprotection.

Microglia and macrophages are the primary immune cells in the brain, rapidly activated after ischemic injury. They exhibit both pro-inflammatory (M1) and anti-inflammatory (M2) phenotypes, participating in inflammatory responses, neural damage, and repair [60]. In ischemic cerebrovascular disease, their metabolic profile shifts significantly from oxidative phosphorylation to glycolysis to meet the energy demands of pro-inflammatory factor synthesis, thereby triggering inflammatory responses and promoting neuronal injury [61]. The research team led by Evanna L. Mills discovered that when macrophages are activated by lipopolysaccharide (LPS) to shift from oxidative phosphorylation to glycolysis, succinate levels rise significantly. The oxidation of succinate, combined with ROS production, induces pro-inflammatory gene expression, thereby driving macrophages toward a pro-inflammatory phenotype polarization [62]. During acute cerebral ischemia, succinate also markedly accumulates in the ischemic area. Upon reperfusion, succinate undergoes rapid oxidation, triggering a burst of mitochondrial ROS that further exacerbates tissue damage. Thus, targeting succinate metabolism can effectively block ROS bursts during the reperfusion phase and mitigate ischemia-reperfusion injury [63]. At the same time, it has been found that inhibition of glycolysis by 2-deoxyglucose (2DG) in macrophages can reduce the production of pro-inflammatory cytokines, such as IL-1β, induced by LPS [64]. Consequently, glycolytic blockade reshapes the inflammatory phenotype of macrophages or microglia, ultimately regulating neuroinflammation and preventing secondary neuronal damage.

Overall, the survival of neurons is highly dependent on the metabolic support provided by astrocytes through the transport of lactate and glycogen. Such energy substrates can alleviate the energy crisis of neurons under ischemia or high metabolic stress. Meanwhile, inhibition of glycolysis in microglia and infiltrating macrophages can attenuate neuroinflammation and promote tissue repair by affecting their inflammatory phenotype switching. This mechanism of metabolic-immune co-regulation is essential for maintaining neuronal function and survival.

## Lactate in Ischemic Cerebrovascular Disease

The above discussion highlights the pivotal role of glycolysis in the pathophysiology of ischemic cerebrovascular disease. As the end product of glycolysis, lactate also has multifaceted roles, including its traditional function as a metabolic product and emerging roles in lactylation regulation and signal transduction.

### Traditional Functions of Lactate

As previously mentioned, during ischemic cerebrovascular disease, reduced blood supply to the brain leads to insufficient oxygen delivery. This drop in intracellular oxygen pressure limits mitochondrial aerobic respiration, forcing cells to rely on anaerobic metabolism for energy generation. Glucose is metabolized into pyruvate *via* glycolysis, and pyruvate is reduced to lactate under hypoxic conditions, producing a small amount of ATP. Although this process yields less energy, it can rapidly provide energy in hypoxic environments, sustaining basic cellular functions. Thus, lactate production in the early stages of ischemia is an emergency response to meet energy demands. The generated lactate provides a limited but critical energy source for brain cells, helping to maintain essential physiological functions [65], such as ion pump activity, and the synthesis and release of neurotransmitters. Carole Berthet’s team found that lactate exhibits a dual role in ischemic brain injury models. Specifically, at low concentrations, lactate exerts protective effects by supporting energy metabolism and mitigating cellular stress, whereas at high concentrations, it may exacerbate tissue damage [66]. In a mouse model of transient middle cerebral artery occlusion (MCAO), lactate injection provides protective effects lasting up to two weeks post-ischemia, significantly reducing brain atrophy and neurological deficits [67]. Furthermore, systemic intravenous lactate administration is also effective, making it more feasible for clinical applications [67]. Overall, moderately increasing lactate levels following ischemia may serve as a potential therapeutic strategy, particularly during the early stages of reperfusion.

**Fig. 4:**
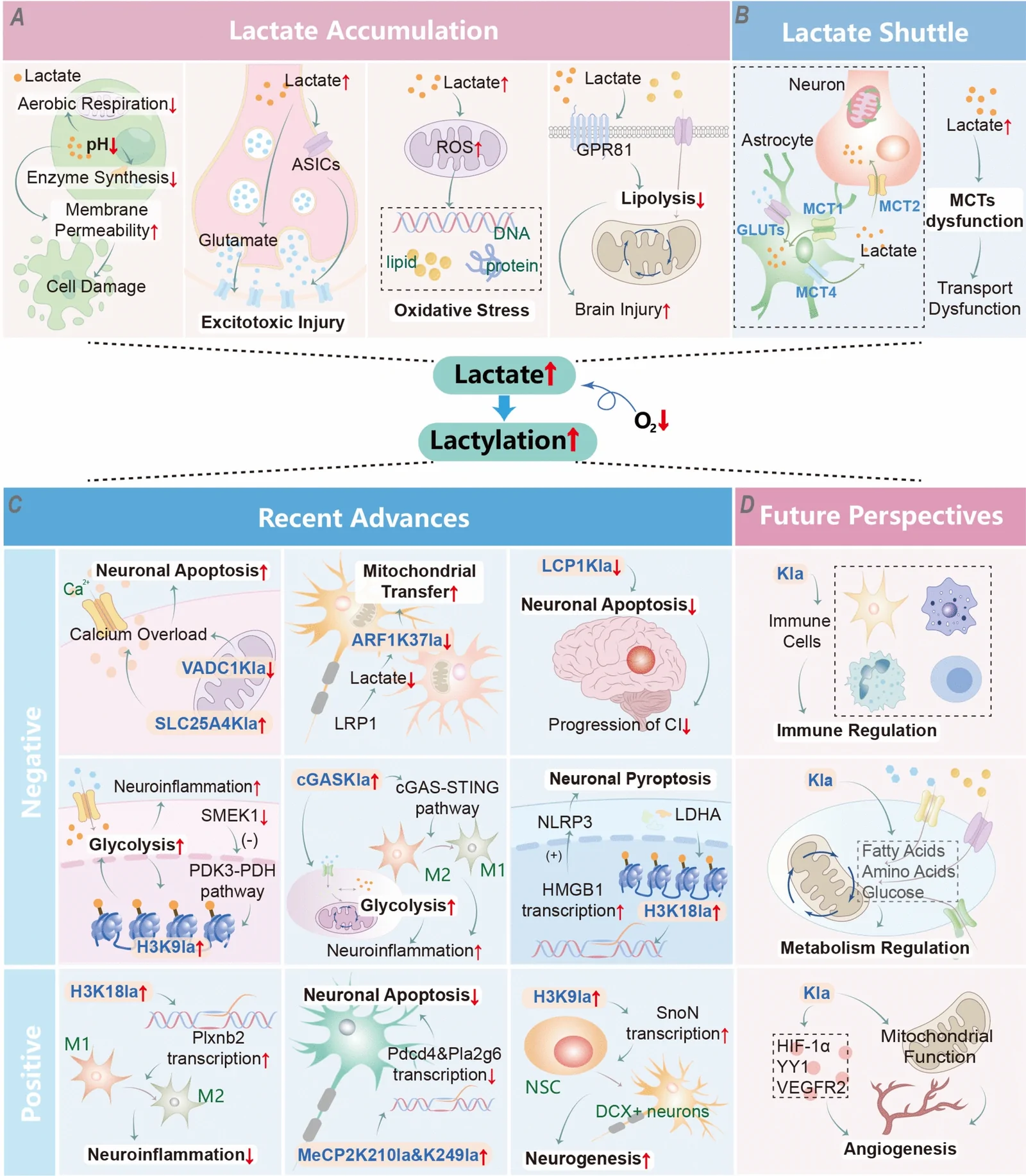
The roles of lactate and lactylation in ischemic cerebrovascular diseases. Under ischemic and hypoxic conditions, the level of lactate in brain tissue increases. leading to acidosis, oxidative stress, and neuronal excitotoxic injury. Elevated lactate also disrupts the astrocyte-neuron lactate shuttle, impairing metabolic support to neurons. Moreover, lactate functions as a signaling molecule, further influencing cellular responses during ischemic injury. Elevated lactate levels in brain tissue result in an increase in protein lactylation. Lactylation exerts multifaceted effects in ischemic cerebrovascular diseases by regulating key pathological processes, such as neuronal apoptosis (targeting PDK3-PDH pathway, cGAS-STING pathway and plxnb2), neuroinflammation (targeting VADC, SLC25A4, LCP1, MeCP2, pdcd4 and pla2g6), pyroptosis (targeting HMGB1 and NLRP3), glycolysis (targeting PDK3-PDH pathway and cGAS-STING pathway), mitochondrial function (targeting ARF1), etc. Additionally, lactylation is anticipated to provide new therapeutic insights for ischemic cerebrovascular disease by modulating neuroinflammation, metabolic regulation, and angiogenesis. Symbols: (+) = Activation, (-) = Inhibition.

However, in prolonged ischemia, excessive lactate accumulation leads to intracellular acidosis. A significant drop in intracellular pH disrupts normal cellular metabolic processes [66], including mitochondrial aerobic respiration, protein synthesis, and enzyme activity. Numerous studies have shown that lactic acidosis activates acid-sensing ion channels (ASICs) or triggers glutamate release [68], thereby promoting excitotoxic neuronal injury [68–70]. Additionally, acidosis increases cell membrane permeability, disrupting intracellular ion balance and further exacerbating cellular damage [71]. Simultaneously, lactate accumulation triggers oxidative stress. Under ischemic conditions, despite insufficient oxygen supply, the cell must still maintain redox balance. Lactate accumulation increases the NADH/NAD⁺ ratio, inhibiting the mitochondrial electron transport chain and enhancing ROS generation [72, 73]. In neurons, excess lactate further stimulates ROS production, which disrupts mitochondrial energy metabolism and drives oxidative stress. This, in turn, impairs ATP synthesis, creating a vicious cycle of ROS overproduction. Excessive ROS damages cellular macromolecules, including lipids, proteins, and DNA, ultimately leading to oxidative injury [74] (Fig. 4A).

### Lactate Transport

To gain a deeper understanding of lactate’s functions, it is also essential to grasp the lactate shuttle theory. Monocarboxylate transporters (MCTs), primarily located in red blood cells, play a critical role in the transmembrane transport of lactate [75]. Later studies revealed that MCTs are also expressed on the membranes of various cells and organelles [76]. This discovery laid the foundation for the lactate shuttle theory, which posits that lactate can be transported *via* MCTs from regions of high concentration to regions of low concentration [77].

To date, 14 MCT subtypes have been identified, with MCT1, MCT2, and MCT4 being the most functionally characterized [76]. Astrocytes predominantly express MCT1 and MCT4 [78]. MCT4 facilitates lactate efflux from intracellular compartments, while MCT1 mediates lactate influx from the extracellular environment, with the lactate concentration gradient serving as the key determinant of transport directionality [79, 80]. In contrast, MCT2 is predominantly localized in neurons. Lactate can enter neurons *via* MCT2 and be oxidatively utilized, thereby providing energy support for neuronal activity [81]. As previously described, astrocytes emerge as the primary ​bioenergetic hubs in cerebral tissue during hypoxia. Through accelerated glycolysis, astrocytes generate lactate, which is subsequently shuttled to neurons *via* MCTs. Within neurons, lactate undergoes conversion into pyruvate to fuel the TCA cycle and oxidative phosphorylation (OXPHOS), thereby sustaining neuronal metabolic homeostasis and physiological function. Collectively, the astrocyte-neuron lactate shuttle plays a critical role in ischemic cerebrovascular disease.

During cerebral ischemia, the function of MCTs is also impaired. Ischemia disrupts energy metabolism, leading to ATP reduction and impaired maintenance of proton gradients and membrane potential, thereby compromising the normal transport function of MCTs [82]. Inhibition of MCTs prevents efficient lactate release from astrocytes, resulting in the intracellular accumulation of lactate. Consequently, restricted astrocytic lactate export compels neurons to depend on their own anaerobic metabolism, thereby aggravating the energy crisis [83]. Simultaneously, the inability of MCTs to transport lactate into neurons increases extracellular lactate accumulation. This accumulation may further exacerbate extracellular acidosis, thereby activating ASICs and leading to neuronal damage [84, 85]. Studies have shown that ASIC activation induces Ca^2^⁺ influx, markedly elevating intracellular Ca^2^⁺ levels. This, in turn, activates multiple Ca^2^⁺-dependent enzymes, leading to biomolecule degradation and ultimately triggering neuronal apoptosis or necrosis [84] (Fig. 4B).

### Emerging Functions of Lactate

Based on the lactate shuttle theory, recent studies have revealed that lactate is not merely a byproduct of anaerobic metabolism but also serves as a crucial signaling molecule. It has been shown to activate cell surface receptors, such as the hydrogen ion-gated receptor GPR81, a G protein–coupled receptor (GPCR) originally identified in adipocytes [86]. Later studies found that GPR81 is also expressed in tumor cells and immune cells [87, 88]. Lactate, as an endogenous ligand of GPR81, activates this receptor to inhibit cyclic adenosine monophosphate (cAMP) levels and protein kinase A (PKA) activity [89, 90], promoting energy metabolite storage and inhibiting lipolysis [90]. This enhances neuronal vulnerability to ischemia, leading to cell death and expansion of the infarct area [56, 91] (Fig. 4A).

Beyond its role as a metabolic byproduct and signaling mediator, lactate’s emerging critical role lies in its ability to initiate lactylation—a process highlighted in this review as the “glycolysis-lactate-lactylation” axis. The following sections will detail the implications of lactate-driven lactylation in the pathogenesis and potential treatment of ischemic cerebrovascular disease.

## Lactylation in Ischemic Cerebrovascular Disease

As described above, in ischemic cerebrovascular disease, the upregulation of glycolysis in brain tissue leads to significant lactate accumulation. Elevated lactate level enhances the activity of lactylation and participates in PTMs of multiple proteins (Fig. 4C).

### Current Progress

A growing body of research on lactylation in ischemic cerebrovascular disease has emerged, clearly demonstrating a complex dual role of lactylation in disease pathogenesis.

Lactylation exacerbates brain injury by inducing neuronal apoptosis. In 2022, a research team discovered that in a cerebral ischemia-reperfusion injury (CIRI) rat model, lysine lactylation (Kla) of mitochondrial membrane proteins SLC25A4 (K245) and SLC25A5 (K96) was significantly upregulated in brain endothelial cells, while Kla of VDAC1 (K20, K266) was eliminated. These proteins are involved in the Ca^2^⁺ signaling pathway, and their aberrant lactylation disrupts Ca^2^⁺ homeostasis, leading to Ca^2^⁺ overload, mitochondrial dysfunction, and neuronal apoptosis [92]. Beyond impairing mitochondrial function, lactylation can also modulate apoptosis by affecting mitochondrial transfer. In ischemic stroke, astrocytes release healthy mitochondria to support the survival and functional recovery of damaged neurons [29]. A recent study found that low-density lipoprotein receptor-related protein 1 (LRP1) lowers astrocytic lactate production and ADP-ribosylation factor 1 (ARF1) lactylation, thereby increasing mitochondrial release and astrocyte-to-neuron transfer. This, in turn, reduces neuronal apoptosis, improves neurological function after ischemia-reperfusion, and mitigates ischemic organ injury [29]. More notably, the study includes observational analyses of clinical samples, revealing significantly elevated cerebrospinal fluid (CSF) lactate levels in patients with stroke compared to normal controls. Correlation analyses further demonstrate that CSF lactate concentrations are positively associated with infarct volume but inversely correlate with the abundance of astrocyte-derived mitochondria in the CSF. These clinical findings corroborate observations from mouse models, indicating that lactate impairs functional mitochondrial transfer and exacerbates ischemia–reperfusion injury [29]. Additionally, studies have demonstrated that the lactylation level of lymphocyte cytoplasmic protein 1 (LCP1) is markedly upregulated in both a rat model of MCAO and PC12 cells subjected to oxygen-glucose deprivation/reoxygenation (OGD/R). Lactylation of LCP1 enhances the protein stability of LCP1, thereby prolonging its intracellular presence and increasing its functional activity. Notably, the increased level of LCP1 can not only promote cell apoptosis but also disrupt cellular energy metabolism, thereby exacerbating hypoxia-induced cell injury and contributing to the progression of cerebral infarction (CI). The study also shows that treatment with the glycolysis inhibitor 2-Deoxy-D-glucose (2-DG) significantly reduces LCP1 lactylation, decreases apoptosis, and improves neuronal survival [93]. This not only validates 2-DG as a metabolic intervention that confers neuroprotection in cerebral-infarct models, but more importantly, elegantly links metabolic status, post-translational protein modification, and cell fate, thereby offering fresh insights and potential therapeutic targets for ischemic stroke. It should be emphasized, however, that 2-DG was employed here as a mechanistic probe, and substantial challenges related to selectivity, side effects, and other safety concerns remain to be addressed before its clinical translation [93].

Moreover, lactylation further aggravates brain injury by reprogramming cellular metabolism and amplifying neuroinflammatory responses. Firstly, abnormal energy metabolism drives the occurrence of lactylation, while lactylation reciprocally modulates dysregulated metabolic pathways. In Alzheimer’s disease (AD) research, it was found that lactate accumulation induces lactylation of glycolysis-related proteins, such as HIF-1α, PKM2, and lactate dehydrogenase A (LDHA). The lactylation of these proteins further enhances glycolysis, creating a positive feedback loop that perpetuates neuronal reliance on glycolysis, exacerbating metabolic imbalance and lactate accumulation [94]. A similar mechanism exists in ischemic cerebrovascular disease. Buyang Huanwu Decoction (BHD), a traditional Chinese formula widely used in the clinical treatment of ischemic cerebrovascular disease, exerts its neuroprotective effects through lactylation-mediated epigenetic modifications and regulation of the Apaf-1 apoptosis pathway, providing new insights into the modern mechanisms underlying traditional formulations [95]. Specifically, BHD suppresses key glycolytic phenotypes and downregulates pan-lysine lactylation (pan-Kla) and histone H3K18 lactylation (H3K18la), thereby disrupting the glycolysis-lactylation positive feedback loop. It also downregulates Apaf-1 transcriptional activity, thereby reducing apoptosis in brain microvascular endothelial cells [95]. Another study reveals a similar mechanism, demonstrating that the sterile alpha motif and EGF-like domain 1 (SMEK1) affects neuroinflammation and brain injury in acute ischemic stroke (AIS) by regulating the histone lactylation-mediated microglial glycolysis-lactate metabolic axis. This work first identifies SMEK1 as a novel regulator of histone lactylation, linking metabolic reprogramming with epigenetic modifications. Microglial-specific SMEK1 deletion inhibits the PDK3-PDH pathway (PDH is pyruvate dehydrogenase, and PDK3 is its kinase), leading to increased H3K9la, epigenetic activation of LDHA and HIF-1α transcription, enhanced glycolysis, and further lactate production, forming a “lactate overload→histone lactylation→enhanced glycolysis” positive feedback loop, thereby exacerbating dysregulation of lactate metabolism and neuroinflammation [96]. It is noteworthy that SMEK1 exhibits dynamic changes in expression between the early and late phases of ischemia. In the early ischemic phase, a transient increase in SMEK1 inhibits lactate production and H3K9la, thereby blocking the initiation of microglial M1 polarization. In contrast, its expression declines sharply in the late phase, accompanied by exacerbated neuroinflammation. This suggests that maintaining or enhancing SMEK1 levels during the early phase represents a critical therapeutic window for preventing a detrimental metabolic‑epigenetic cascade [96].

The impact of lactylation on neuroinflammation and glycolysis has also been corroborated in another pathway. Previous studies have shown that, in cisplatin-induced cochlear hair cell damage models, mitochondrial dysfunction leads to the release of mitochondrial DNA (mtDNA) into the cytoplasm, activating the cGAS-STING pathway, which in turn triggers NF-κB-mediated inflammatory responses and apoptosis [97]. Notably, activation of the cGAS-STING pathway is not limited to auditory system damage but is also critical in ischemic brain injury. The team led by Lisheng Wang found that aberrant lactylation of cyclic GMP-AMP synthase (cGAS) is closely related to its mediating neuronal inflammatory injury. Experiments demonstrated that OGD markedly increases cGAS lactylation in microglia, enhancing cGAS protein stability and causing its aberrant intracellular accumulation. High levels of cGAS persistently activate the downstream cGAS-STING pathway, driving microglia toward the M1 phenotype and amplifying neuroinflammation. Concurrently, this signaling reinforces glycolysis, generating more lactate and ultimately exacerbating brain injury. The revelation of this mechanism positions cGAS lactylation as a critical link between metabolic dysregulation and neuroinflammation, offering a novel therapeutic target for HIE [98]. In addition to microglia, lactylation can also exacerbate neuroinflammation and brain damage by driving the phenotypic switch of astrocytes. Research has revealed that under ischemic stress, glycolysis in astrocytes is enhanced, leading to massive lactate production through the upregulation of LDHA activity. This lactate is taken up into neurons *via* MCT2, where it induces lactylation of specific proteins, thereby directly driving neuronal dysfunction and death. Meanwhile, this process also promotes the release of pro-inflammatory factors from microglia, thereby triggering the transformation of astrocytes toward a neurotoxic A1 phenotype. Ultimately, these two pathological processes act synergistically to exacerbate neuroinflammation and aggravate brain damage [99]. Furthermore, in this study, various interventions targeting different stages of lactylation formation—such as reducing lactate accumulation, blocking lactate transport, inhibiting lactylation “writer” enzymes, and genetic interventions—have been employed to validate the role of lactylation. These measures effectively lower the levels of lactylation in neurons, resulting in reduced neuronal death, decreased glial activation, extended reperfusion time windows, improved functional recovery, and reduced infarct volumes [99]

Notably, lactylation has also been implicated in neuronal pyroptosis, a form of programmed cell death driven by inflammasome activation and characterized by the release of pro-inflammatory cytokines, such as IL-1β and IL-18 [100]. Under ischemia-reperfusion injury, elevated LDHA enhances H3K18la, thereby upregulating HMGB1. Increased HMGB1 activates the NOD-like receptor family pyrin domain-containing 3 (NLRP3) inflammasome, thereby promoting neuronal pyroptosis and ultimately aggravating neuronal damage. Experiments show that LDHA knockdown inhibits histone lactylation enrichment at the HMGB1 promoter region and reduces HMGB1 expression, while lactate supplementation reverses these effects, indicating that LDHA regulates HMGB1 transcription through lactylation [101]. Furthermore, LDHA silencing significantly reduces pyroptosis-related markers, and HMGB1 overexpression reverses this inhibition, confirming HMGB1 as a key downstream effector of LDHA [101].

Although lactylation is generally shown to exacerbate ischemic brain injury in most studies, some studies indicate that elevated lactylation levels may also exert beneficial effects, particularly in the context of neuroinflammation. In a 2025 study, researchers utilized a mouse model of middle cerebral artery occlusion (MCAO) and found that H3K18la levels were significantly elevated in microglia. This histone modification upregulates the expression of Plexin-B2 (Plxnb2) in microglia, promoting their polarization toward the M2 phenotype, which in turn alleviates neuroinflammation and neural damage after ischemic stroke and facilitates neurological recovery. Further experiments demonstrate that specific knockdown of Plxnb2 in microglia reverses the anti‑inflammatory phenotype, dendritic morphological remodeling, and neuroprotective effects induced by lactate [102]. Lactylation can also exert protective effects by modulating gene expression. In a study of ischemic stroke in mice, lactylations are identified at lysine residues K210 and K249 of the non-histone protein MeCP2 in neurons. These modifications suppress the transcription of the pro-apoptotic genes Programmed Cell Death 4 (Pdcd4) and Phospholipase A2 Group VI (Pla2g6), thereby reducing neuronal apoptosis, decreasing infarct volume, and improving neurological function. Specifically, when lactylation is inhibited through methods such as gene knockout or pharmacological intervention, Pdcd4 and Pla2g6 expression increase, accompanied by elevated numbers of apoptotic neurons, enlarged infarct volume, and worsened neurological outcomes. Conversely, exogenous lactate supplementation reverses neuronal damage, further confirming the neuroprotective roles of MeCP2K210la and MeCP2K249la [103].

Beyond the above protective roles, lactylation also contributes to brain injury repair. Research has shown that under hypoxic conditions, lactate increases H3K9la levels in neural stem cells (NSCs), which promotes the expression of the Ski-like oncogene (SnoN). This in turn drives the differentiation of NSCs toward a neuronal lineage, increasing the number of Doublecortin-positive (DCX+) newborn neurons, thereby facilitating neurogenesis and promoting brain injury repair [104].

In summary, current preclinical studies have clearly delineated the dual roles of protein lactylation in ischemic cerebrovascular disease. On one hand, lactate accumulation-driven lysine lactylation exacerbates brain injury by triggering neuronal apoptosis and pyroptosis, disrupting normal metabolism, and amplifying neuroinflammation. On the other hand, lactylation under specific contexts demonstrates neuroprotective potential by suppressing apoptotic gene transcription, fostering an anti-inflammatory microenvironment, and promoting neurogenesis. Crucially, interventions targeting this axis, including p300 inhibition and glycolytic inhibitors, have consistently shown significant neuroprotective effects in animal models, thereby robustly validating the “glycolysis-lactate-lactylation” axis as a viable therapeutic target.

### Future Perspectives

Despite emerging studies on lactylation in ischemic cerebrovascular disease, many of its functional roles remain undiscovered, and current evidence is still limited. In this section, we elaborate on the potential mechanisms through which lactylation may contribute to the pathophysiology of this condition (Fig. 4D).

#### Lactylation and Immune Regulation

Following ischemia, neuroinflammation is a key pathological process driving brain injury and dysfunction [105]. Neuroinflammation involves the activation of endogenous immune cells within the central nervous system (CNS), such as microglia, as well as the infiltration of peripheral immune cells, including macrophages, neutrophils, and T lymphocytes [106]. In recent years, lactylation, as a newly discovered post-translational modification, has been shown to regulate the function of various immune cells, thereby playing a significant role in neuroinflammation.

In addition to the previously described effects of microglial lactylation in ischemic cerebrovascular diseases, macrophages also play a crucial role in neuroinflammation following ischemic stroke [107, 108]. In myocardial infarction models, peripheral macrophages acquire a reparative phenotype through lactylation, exhibiting both anti-inflammatory and pro-angiogenic effects, which aid tissue repair [109]. Similar mechanisms may exist in ischemic stroke, where lactylation could regulate macrophage phenotypes, impacting their role in neuroinflammation [110]. Additionally, lactylation may influence neuroinflammation progression by modulating HMGB1 release, affecting macrophage chemotaxis and activation [111, 112].

Neutrophils are among the earliest peripheral immune cells to infiltrate the injury site following ischemic stroke [113]. They exacerbate secondary brain injury by secreting pro-inflammatory factors, ROS, and proteases [114]. Studies have shown that elevated lactylation levels enhance the expression of chemokines such as TNF-α, IL-1β, IL-6, and CXCL8A, promoting neutrophil recruitment. Conversely, inhibiting lactylation reduces neutrophil infiltration and alleviates inflammatory responses [115, 116]. Furthermore, lactylation may regulate neutrophil metabolic states, influencing their function in ischemic stroke.

In addition to the above cells, T lymphocytes also play a critical role in the immune response to ischemic cerebrovascular disease [117, 118]. Subpopulations such as Th17 cells, regulatory T cells (Tregs), and natural killer T cells (NKTs) exhibit diverse functions, and lactylation can regulate the differentiation and function of T lymphocytes. For example, lactylation promotes the differentiation of Th17 cells by modifying the IKZF1 protein, influencing their role in inflammation [119]. In the tumor microenvironment, lactylation enhances the stability and function of Tregs, suppressing immune responses [120]. Although direct evidence linking lactylation to T lymphocytes in ischemic stroke is lacking, insights from other disease models suggest that lactylation may influence the distribution and function of T lymphocyte subpopulations, thereby modulating post-stroke neuroinflammation.

Overall, lactylation exerts a profound impact on neuroinflammation by modulating the phenotypes and functionality of diverse immune cells. On this basis, we hypothesize that lactylation may regulate immune cell dynamics, thereby influencing the pathogenesis and progression of ischemic cerebrovascular disease. This mechanistic link positions lactylation as a promising candidate for both investigating disease mechanisms and developing novel therapeutic strategies in ischemic cerebrovascular disorders.

#### Lactylation and Metabolic Regulation

During ischemic stroke, the interruption of cerebral blood flow leads to insufficient oxygen and nutrient supply, triggering metabolic dysregulation, including disturbances in glucose, lipid, and amino acid metabolism [121]. This metabolic imbalance not only exacerbates brain tissue damage but also affects the recovery process. Recent studies have identified lactylation as a critical regulator of diverse metabolic pathways, highlighting its potential significance in the pathogenesis and therapeutic targeting of ischemic cerebrovascular disease [122].

During ischemic stroke, glycolysis becomes the primary pathway for energy production under hypoxic conditions. In the study of macrophages, lactylation can regulate pyruvate kinase M2 (PKM2), a key enzyme in glycolysis [123]. Lactylation of PKM2 enhances its enzymatic activity, promoting glycolysis and providing cells with more energy. However, this enhanced glycolysis may also lead to excessive lactate accumulation, further exacerbating cellular acidosis and damage. In AD, histone lactylation, especially H4K12la, can directly activate the transcription of glycolytic genes, thereby increasing the expression of glycolysis-related enzymes and promoting the glycolytic process [94]. A similar mechanism may also be involved in ischemic cerebrovascular disease.

Lactylation also regulates lipid and amino acid metabolism. Studies have shown that in stroke, fatty acid synthase (FASN)-driven lipogenesis contributes to the repair of the blood-brain barrier by increasing the expression of IL-33, and regulates neuroinflammation to create favorable conditions for neurological recovery [124]. It is reported that lactylation of FASN can inhibit the activity of FASN, and reduce the synthesis of fatty acids, thereby affecting cellular energy metabolism and function [125, 126]. In ischemic stroke models, carnitine palmitoyltransferase 2 (CPT2) activity is markedly reduced, leading to impaired fatty acid oxidation, mitochondrial dysfunction, and disrupted cellular energy supply [127]. Notably, lactylation has been shown to modulate the activity of CPT2, thereby influencing fatty acid metabolism [128]. After ischemic stroke, amino acid metabolism also undergoes significant changes, with increased glutamine breakdown and branched-chain amino acid utilization to meet cellular energy demands [121]. Lactylation may regulate the activity of key enzymes such as glutaminase (GLS), thereby influencing glutamine metabolism [121].

Mitochondrial dysfunction is a central contributor to cellular damage following ischemic stroke. Recent studies in skeletal and cardiac muscle have shown that, during exercise, lactate oxidation lowers intracellular oxygen, activates AARS2, increases PDHA1-K336la and CPT2-K457/8la, and thereby feedback-inhibits mitochondrial metabolism to suppress oxidative phosphorylation and fatty-acid oxidation while potentially protecting cells by curbing ROS. These findings highlight lactylation as a key integrator of hypoxic and metabolic signals that govern mitochondrial function [128]. Given that cerebral ischemia also features marked lactate rise and mitochondrial suppression, this mechanism is likely conserved in neurons or glia and may protect the ischemic brain by preventing excessive energy activation in the early phase of stroke. Additionally, under ischemic conditions, excessive mitochondrial fission disrupts energy homeostasis and promotes cellular damage. Lactylation has been shown to modulate mitochondrial fission protein 1 (Fis1), thereby influencing mitochondrial dynamics. Specifically, by enhancing Fis1 activity, lactylation may aggravate mitochondrial fragmentation and exacerbate energy deficits [129]. In parallel, moderate mitophagy plays a protective role by removing damaged mitochondria and sustaining cellular metabolic balance [130, 131]. Lactylation may also regulate key mitophagy-related proteins, thereby impacting mitophagy efficiency, neuronal survival, and functional recovery after ischemia [132].

As an emerging post-translational modification, lactylation plays a significant role in regulating metabolic processes. On one hand, lactylation may enhance glycolysis and fatty acid synthesis to provide energy for cells, and promote anti-inflammatory responses and functional recovery by regulating metabolic enzyme activity. On the other hand, lactylation may also lead to lactate accumulation and mitochondrial dysfunction, thus exacerbating cellular damage. Further research is needed to comprehensively elucidate the specific mechanisms of lactylation in different metabolic pathways, as well as its spatiotemporal distribution and functional differences in ischemic stroke, providing a theoretical basis for developing novel therapeutic strategies targeting lactylation.

#### Lactylation and Angiogenesis

Beyond the aforementioned mechanisms, emerging evidence suggests that lactylation may also critically regulate angiogenesis, thereby playing a pivotal role in the pathophysiological processes of ischemic cerebrovascular disease. Angiogenesis is a critical process for post-ischemic brain recovery, promoting oxygen and nutrient supply to damaged brain regions, reducing infarct volume, and improving neurological function [133]. Lactylation may regulate factors associated with angiogenesis through multifaceted mechanisms, thereby modulating the pathophysiological progression of ischemic stroke.

First, lactylation can regulate the stability of HIF-1α, a key transcription factor promoting angiogenesis under hypoxic conditions [134]. In prostate cancer models, lactylation of HIF-1α enhances its stability, preventing its degradation in normoxic environments, and upregulates the expression of vascular endothelial growth factor A (VEGFA), promoting angiogenesis [27]. Lactylation can also regulate other angiogenesis-related proteins, such as YY1 and vascular endothelial growth factor receptor 2(VEGFR2). YY1 is a multifunctional transcription factor, and studies have found that lactylation enhances its binding to the FGF2 promoter, thereby promoting retinal neovascularization [135]. VEGFR2, the receptor for VEGFA [136], can be activated by lactylation, thereby promoting angiogenesis [137]. However, lactylation may also inhibit angiogenesis through other pathways, such as suppressing semaphorin 3A expression *via* histone H3K18la modification [138]. Additionally, lactylation can influence endothelial-mesenchymal transition (EndMT) by regulating the TGF-β/Smad2 pathway, further impacting angiogenesis [139]. Moreover, as previously mentioned, lactylation regulates mitochondrial function and metabolic states, which can indirectly affect angiogenesis.

In summary, lactylation may regulate angiogenesis through multiple mechanisms, including modulating the activity and stability of key factors such as HIF-1α, YY1, and VEGFR2, as well as influencing mitochondrial function and metabolism. These findings underscore the important role of lactylation in the pathophysiology of ischemic stroke. Future research is needed to further elucidate the specific mechanisms of lactylation in angiogenesis, providing new therapeutic targets for ischemic stroke.

## Clinical Trials on Lactylation

**Fig. 5:**
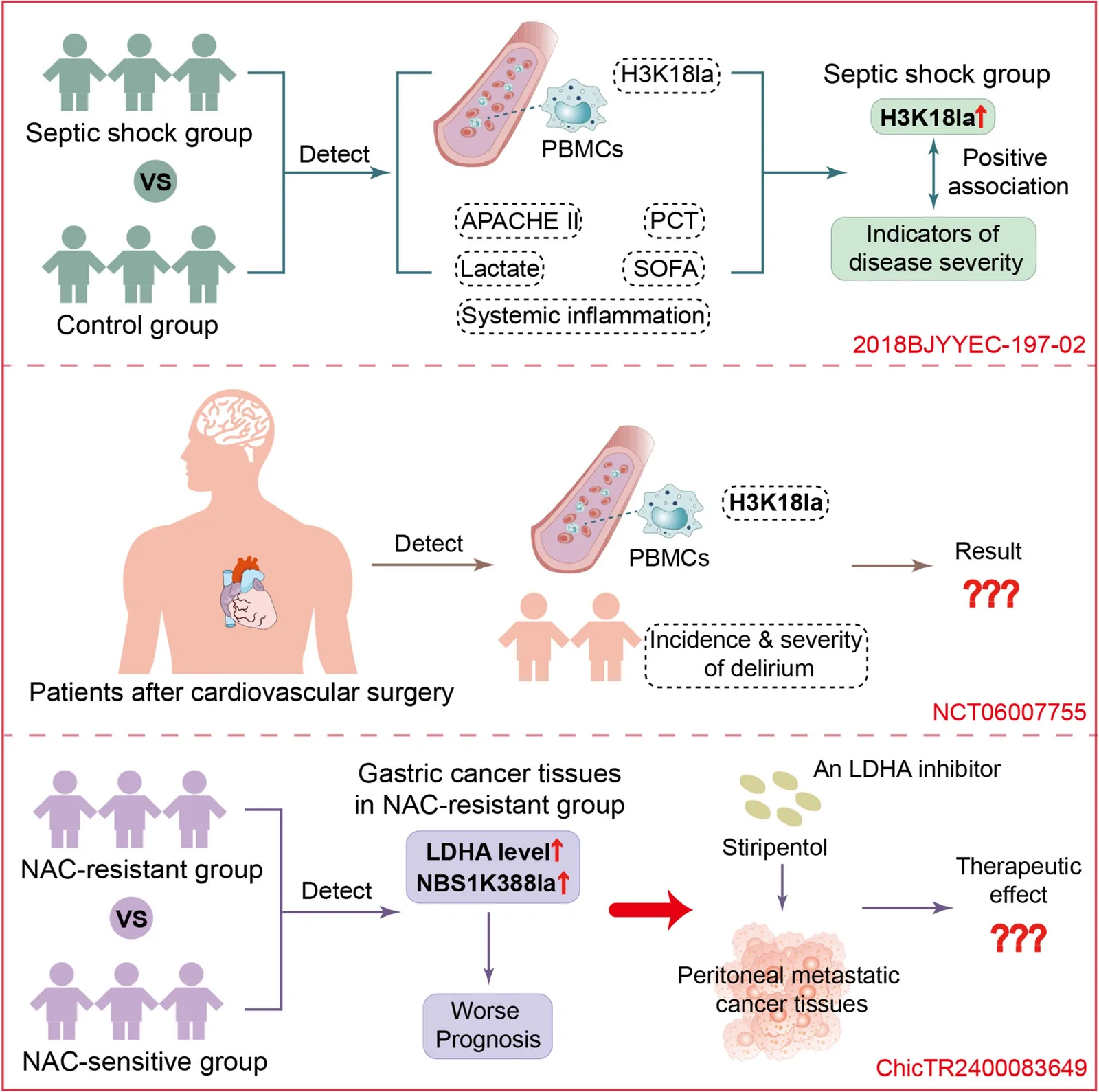
Clinical implications of lactylation. (Top) In this clinical trial (2018BJYEC-197-02), patients with septic shock exhibit significantly elevated H3K18la levels in PBMCs, which are positively correlated with disease severity indicators, including APACHE II and SOFA scores, serum lactate, PCT, and systemic inflammatory markers. (Center) An ongoing clinical trial (NCT06007755) investigates monocyte H3K18la levels in patients after cardiovascular surgery, exploring its association with postoperative delirium incidence and severity. (Bottom) A retrospective analysis has shown that in gastric cancer tissue of NAC-resistant patients, both NBS1 K388la and LDHA expression are significantly increased. A phase II clinical trial (ChiCTR2400083649) is currently underway to evaluate stiripentol, an LDHA inhibitor, as a therapeutic agent for patients with peritoneal metastatic cancer. Abbreviations: NAC, Neoadjuvant Chemotherapy.

As detailed in the preceding sections, the landscape of lactylation research in ischemic cerebrovascular disease reveals its profound dual roles and therapeutic potential. However, direct clinical evidence targeting this novel modification remains in its nascent stages. Preclinical models have robustly demonstrated that interventions at various nodes of the “glycolysis-lactate-lactylation” axis, such as inhibiting key enzymes like p300, or utilizing glycolysis inhibitors such as 2-deoxyglucose, can effectively mitigate neuronal death, neuroinflammation, and infarct volume by modulating protein lactylation. These studies establish a compelling cause-and-effect relationship between lactylation and pathological outcomes, providing a solid mechanistic foundation for therapeutic targeting. Nevertheless, the translation of these preclinical insights into human applications is just commencing. The following section will summarize the emerging clinical trials investigating lactylation, primarily in other disease contexts, which nonetheless highlight the feasibility of measuring lactylation in patient samples and hint at its broader clinical relevance (Fig. 5). These pioneering efforts lay the groundwork for future clinical research specifically designed to validate the diagnostic, prognostic, and therapeutic significance of lactylation in ischemic cerebrovascular disease.

In 2022, a historical cohort study examined the expression level of H3K18la in peripheral blood mononuclear cells (PBMCs) of patients with septic shock, and its correlation with disease severity and prognosis (the ethical approval number is 2018BJYEC-197-02) [140]. The results demonstrated a significant elevation of H3K18la level in PBMCs from patients with septic shock compared to healthy controls. Crucially, H3K18la expression exhibited a positive correlation with established indicators of disease severity, including the Acute Physiology and Chronic Health Evaluation II (APACHE II) score, the Sequential Organ Failure Assessment (SOFA) score, serum lactate levels, infection markers such as procalcitonin (PCT), and systemic inflammation [140]. These findings position H3K18la as a potential diagnostic and prognostic biomarker for septic shock. Nevertheless, given the limitations of the initial study, including a small sample size and single-center design, these promising results warrant validation in larger, multicenter cohorts.

In 2023, Huazhong University of Science and Technology conducted a prospective cohort study on postoperative delirium following cardiovascular surgery (NCT06007755). As mentioned above, the expression level of H3K18la in PBMCs has been significantly associated with disease severity and inflammation [140]. Since inflammation also plays a crucial role in delirium after cardiovascular surgery, this study speculates that the level of H3K18la in monocytes may be related to postoperative delirium. The study is designed to measure serum monocyte H3K18la levels and their correlation with the incidence and severity of postoperative delirium in patients undergoing major cardiac surgery. The results of the research have not been announced yet.

It is worth noting that clinical investigations targeting lactylation-related enzymes have also been initiated. In animal experiments, researchers have identified stiripentol, an LDHA inhibitor, as a compound that reduces Nijmegen breakage syndrome 1 (NBS1) lactylation by lowering lactate levels, thereby impairing DNA repair of cancer cells and sensitizing gastric cancer cells to chemotherapy [12]. In their study, retrospective analysis of patient samples revealed that elevated NBS1 K388 lactylation and LDHA expression in tumor tissues of gastric cancer patients were positively associated with chemotherapy resistance and poorer survival. Notably, chemotherapy itself appeared to further elevate tumor LDHA and NBS1 lactylation levels, suggesting therapy-induced metabolic adaptation [12]. These findings support the potential of targeting NBS1 lactylation to enhance the efficacy of chemotherapy and serve as a theoretical basis for the ongoing Phase II trial (ChiCTR2400083649) in patients with peritoneal metastatic cancers.

Although the pathogenic roles and therapeutic potential of lactylation are being increasingly recognized across a range of diseases, direct clinical evidence targeting this novel post-translational modification remains in its infancy, and current research faces multiple bottlenecks. Firstly, the widely cited p300/CBP inhibitors suffer from poor brain penetration, limiting their access to the CNS, and exhibit off-target inhibition of acetyltransferases and other epigenetic enzymes, constraining their application in CNS indications such as ischemic stroke [141, 142]. There is an urgent need for structure-guided design of next-generation allosteric inhibitors with higher selectivity and blood-brain barrier permeability, or for leveraging nano-delivery and pro-drug strategies to enhance CNS exposure. In parallel, selective lactylation “erasers” that directly reverse the modification are also emerging as a promising therapeutic avenue [12]. However, these tools are still at the proof-of-concept stage, and their safety margins and spatiotemporal controllability remain to be defined. More pressing still, the search for clinically actionable biomarkers is hampered by a shortage of high-affinity, single-residue-specific anti-lactylation antibodies and by the absence of tracer probes capable of quantifying global “pan-Kla” burden. Consequently, measurements of hot-spot sites such as H3K18la vary widely among laboratories and show poor reproducibility. While earlier studies have preliminarily demonstrated that lactylation levels can be safely detected in patient samples and correlate with disease severity or therapeutic response, these trials suffer from evident design limitations, such as small sample sizes, lack of independent multicenter validation, overly simple control arms that cannot adequately exclude confounders, and the absence of a unified, standardized quantification protocol, all of which restrict comparability and reproducibility. Moreover, most existing investigations have focused on peripheral blood mononuclear cells or tumor biopsies, and it remains unclear whether CNS-specific lactylation dynamics can be reliably mirrored by peripheral indicators. In summary, translating lactylation from “mechanistic discovery” to “clinical utility” will require rigorous, large-scale, multicenter, longitudinal trials to establish its true diagnostic, prognostic, and pharmacodynamic value.

## Discussion

The pathological mechanisms underlying ischemic cerebrovascular disease are complex, with increasing research attention focused on the interplay between metabolic reprogramming and epigenetic regulation. Our review systematically describes the multiple roles of the “glycolysis-lactate-lactylation” axis in ischemic cerebrovascular disease. Under ischemia and hypoxia conditions, glycolysis activation can temporarily maintain the energy supply of cells, but excessive accumulation of lactate leads to acidosis, oxidative stress, and inflammation, which aggravate neuronal injury. The accumulation of lactate also markedly increases the level of lactylation. As a newly identified post-translational modification, lactylation is widely involved in gene expression, metabolic reprogramming, and immune regulation by regulating the functions of both histone and non-histone proteins.

The current study highlights the dual role of lactylation: on one hand, it significantly exacerbates brain injury by inducing neuronal apoptosis and pyroptosis, disrupting mitochondrial function, aggravating neuroinflammation, and impacting metabolism; on the other hand, under specific conditions, it can suppress pro-apoptotic gene transcription, promote microglial polarization toward the anti-inflammatory M2 phenotype, and drive neurogenesis, thereby exerting neuroprotective and reparative functions. However, the spatiotemporal specificity of protein lactylation in cerebral ischemia, along with the dynamic regulatory mechanisms of its associated modifying and demodifying enzymes, has not been fully elucidated. The existing studies are mostly based on animal models, and the clinical transformation needs to be further verified. In addition, the synergistic effect of lactylation with other modifications, such as acetylation and methylation, and its role in the breakdown of the blood-brain barrier and dysfunction of the neurovascular unit, still need to be explored. Overall, the “glycolysis-lactate-lactylation” axis plays a pivotal role in the pathogenesis of ischemic cerebrovascular disease, and targeting this axis may offer a promising therapeutic strategy for ischemic stroke in the future.
